# Sensitivities of an endemic, endangered California smelt and two non-native fishes to serial increases in temperature and salinity: implications for shifting community structure with climate change

**DOI:** 10.1093/conphys/coy076

**Published:** 2019-02-18

**Authors:** Brittany E Davis, Dennis E Cocherell, Ted Sommer, Randall D Baxter, Tien-Chieh Hung, Anne E Todgham, Nann A Fangue

**Affiliations:** 1 Department of Wildlife, Fish and Conservation Biology, University of California Davis, Davis, CA, USA; 2 Department of Animal Sciences, University of California Davis, Davis, CA, USA; 3 California Department of Water Resources, Division of Environmental Services, PO Box 942836, Sacramento, CA, USA; 4 California Department of Fish and Wildlife, Bay-Delta Region 3, 2109 Arch-Airport Rd., Suite 100, Stockton, CA, USA; 5 Department of Biological and Agricultural Engineering, University of California, Davis, CA, USA

**Keywords:** climate change, Delta Smelt, Largemouth Bass, Mississippi Silverside, salinity, temperature

## Abstract

In many aquatic systems, native fishes are in decline and the factors responsible are often elusive. In the San Francisco Estuary (SFE) in California, interactions among native and non-native species are key factors contributing to the decline in abundance of endemic, endangered Delta Smelt (*Hypomesus transpacificus*). Climate change and drought-related stressors are further exacerbating declines. To assess how multiple environmental changes affect the physiology of native Delta Smelt and non-native Mississippi Silverside (*Menidia beryllina*) and Largemouth Bass (*Micropterus salmoides*), fishes were exposed to serial exposures of a single stressor (elevated temperature or salinity) followed by two stressors (elevated temperature and salinity) to determine how a single stressor affects the capacity to cope with the addition of a second stressor. Critical thermal maximum (CTMax; a measure of upper temperature tolerance) was determined after 0, 2, 4 and 7 days following single and multiple stressors of elevated temperature (16°C vs. 20°C) and salinity (2.4 vs. 8–12 ppt, depending on species). Under control conditions, non-native fishes had significantly higher CTMax than the native Delta Smelt. An initial temperature or salinity stressor did not negatively affect the ability of any species to tolerate a subsequent multiple stressor. While elevated salinity had little effect on CTMax, a 4°C increase in temperature increased CTMax. Bass experienced an additive effect of increased temperature and salinity on CTMax, such that CTMax further increased under multiple stressors. In addition, Bass demonstrated physiological sensitivity to multiple stressors demonstrated by changes in hematocrit and plasma osmolality, whereas the physiology of Silversides remained unaffected. Non-native Bass and Mississippi Silversides showed consistently higher thermal tolerance limits than the native Delta Smelt, supporting their abundance in warmer SFE habitats. Continued increases in SFE water temperatures predicted with climate change may further impact endangered Delta Smelt populations directly if habitat temperatures exceed thermal limits.

## Teaser

This study showed that in three quite different fish species from the San Francisco Estuary, stress from elevated temperature or salinity did not reduce their ability to cope with subsequent stressors.

## Introduction

**Figure 1:**
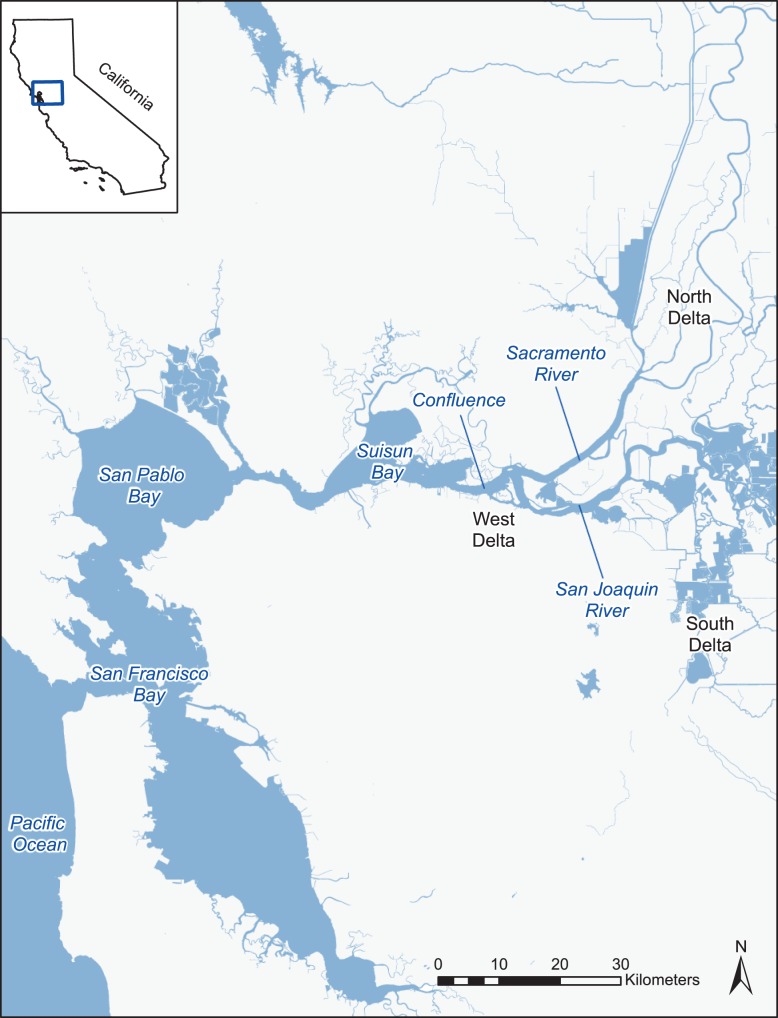
Map of the San Francisco Estuary (SFE) in California, USA. The SFE includes brackish habitats such as San Francisco, San Pablo, and Suisun Bays as well as the freshwater tidal Delta system formed by the confluence of the Sacramento and San Joaquin Rivers.

Climate change is projected to have cascading effects on estuarine and freshwater ecosystems. In addition to rising water temperature and sea level (IPCC, 2013), predicted increases in extreme weather events with climate change are already occurring in some locations. In particular, increased frequency and duration of drought periods are occurring in California and may exacerbate the warming effects of climate change on the San Francisco Estuary (SFE), which includes San Francisco Bay and a tidal freshwater Delta complex formed by the confluence of Sacramento and San Joaquin Rivers (see map of the SFE in Fig. 1). Already, drought periods in the SFE have shown associated increases in water temperature (Jeffries *et al.*, 2016), and changes in salinity regimes may be correlated (Cloern *et al.*, 2011). Although precipitation in California is not consistently predicted to decrease (Dettinger *et al.*, 2015), and may even increase with warming (Polade *et al.*, 2017), the greater incidence of rainfall versus snowfall means that summertime conditions of the SFE are likely to have lower outflows, from decreased influence of snow melt, and increased salinity. Increased temperature and salinity regimes in the SFE ecosystem may lead to negative impacts on biological communities (Knowles and Cayan, 2002; Cloern *et al.*, 2011; Cloern and Jassby, 2012; Mahardja *et al.*, 2017). Already, entire SFE fish assemblages have declined in abundance (a trend called the Pelagic Organism Decline [POD]) including native Osmerids, with some to near extinction (Brown and Moyle, 2005; Feyrer *et al.*, 2007; Sommer *et al.*, 2007; Thomson *et al.*, 2010; Hobbs *et al.*, 2017). While some of the declines have been attributed to impacts to food web dynamics caused by the invasive overbite clam (Feyrer *et al.*, 2003; Mac Nally *et al.*, 2010), non-native predators (Baerwald *et al.*, 2012; Schreier *et al.*, 2016), and physical changes including altered hydrologic regimes (Brown and Bauer, 2010) and increasing water clarity (Mac Nally *et al.*, 2010), it remains unclear how multiple stressors of climate change, such as elevated temperature and salinity interact to affect fish survival and in doing so, influence population abundance and distribution. SFE inflow is highly regulated seasonally for economic uses and maintaining fish habitat, creating substantial conflicts over resource use (Service, 2007; Moyle *et al.*, 2018). Therefore, a better understanding of fish vulnerability to elevated temperature and elevated salinity may provide insight into some of how best to balance conservation efforts for California’s native fishes while also managing the state’s water supply (Brown *et al.*, 2013).

Changes in water temperature and salinity can have profound influences on fish physiology (Fry, 1971; Farrell, 2011). SFE fishes are often exposed to temperatures and salinities outside of their optimal performance range (Brown *et al.*, 2016), and these deviations may result in increased energetic costs due to alterations in cellular physiology, damage to macromolecules, or recruitment of stress response mechanisms (Hasenbein *et al.*, 2013; Sokolova, 2013). For example, high water temperature can affect the biochemistry and physiology of fishes by disrupting macromolecular structures as well as altering rates of metabolic processes (Fry, 1971; Hochachka and Somero, 2002; Fangue *et al.*, 2009). Elevated temperature has also been shown to impact osmoregulatory capacity (Jeffries *et al.*, 2011, 2012), and elevated salinity can cause osmoregulatory imbalance and activate the whole-organism stress response (McCormick *et al.*, 1989; McCormick, 1996; Hasenbein *et al.*, 2013). The combined effects of multiple stressors of elevated temperature and salinity may create competing energetic demands that could affect reproductive fitness and survival. This is particularly relevant for SFE species that have life-history stages that move to different areas of the system, with different temperature and salinity profiles, during ontogeny such as anadromous salmonids or the semi-anadromous Delta Smelt.

One fish of particular concern in the SFE is the Delta Smelt (*Hypomesus transpacificus*). Delta Smelt are a pelagic, semi-andaromous, annual Osmerid endemic to the SFE. While once abundant in the system, the population has declined to critically low levels despite conservation efforts and listing under both state and federal Endangered Species Acts (USFWS 1993, 2010; Sommer and Mejia, 2013; Hobbs *et al.*, 2017). Wild Delta Smelt have been found at temperatures from 6 to 25°C and salinities of 0 to 18 ppt (Moyle, 2002; Nobriga *et al.*, 2008; Sommer and Mejia, 2013); however, they are most frequently found <22°C and <6 ppt (Bennett, 2005; Feyrer *et al.*, 2007, 2013; Sommer and Mejia, 2013), suggesting that temperatures and salinities outside of these ranges may lead to suboptimal physiological performance. Several studies have described the physiological sensitivity of different life stages of Delta Smelt to increased temperatures or salinities of 18–20°C and 4–10 ppt (as single stressors; Swanson *et al.*, 2000; Jeffries *et al.*, 2016; Kammerer *et al.*, 2016; Komoroske *et al.*, 2014, 2015, 2016); however, no study has investigated the interaction of elevated temperature and salinity together on physiological performance of Delta Smelt. Warming has been shown to increase physiological costs of Delta Smelt through increased metabolic rates and upregulation of genes associated with cellular stress response mechanisms (Jeffries *et al.*, 2016; Komoroske *et al.*, 2015, 2016). Increased energetic demands to cope with warming may reduce energy available to cope with a secondary co-occurring stressor such as salinity, leading to potential trade-offs in energy allocation that could impact processes such as osmoregulation. Therefore, projected global climate change conditions (i.e. elevations in both temperature and salinity) may have direct effects on physiological and fitness parameters (i.e. energy allocation, growth, reproduction) of Delta Smelt and substantially affect extinction risk (Cloern *et al.*, 2011; Moyle *et al.*, 2018).

The SFE is one of the most invaded aquatic ecosystems in the world (Mooney and Zavaleta, 2016), creating complex communities of native and non-native species. Although Delta Smelt are endangered and in rapid decline, two non-native species, Mississippi Silversides (*Menidia beryllina*) and Largemouth Bass (*Micropterus salmoides*) are found in significant abundance with increasing distribution ranges (Conrad *et al.*, 2016; Mahardja *et al.*, 2016). Both species influence SFE native fishes through competition for resources and predation (Nobriga *et al.*, 2005). Despite occupying varied habitats, each species has been shown to predate on Delta Smelt (Baerwald *et al.*, 2012; Ferrari *et al.*, 2014; Schreier *et al.*, 2016). While both non-native species are highly eurythermal (Smith and Scott, 1975; Lutterschmidt and Hutchison, 1997; Currie *et al.*, 1998; Swanson *et al.*, 2000; Interagency Ecological Program *et al.*, 2018a, 2018b), they differ in salinity tolerance. Mississippi Silversides are euryhaline (0–35 ppt, Pillard *et al.*, 1999; Interagency Ecological Program *et al.*, 2018a), while Largemouth Bass require low salinities (0–4 ppt in the SFE as described in Conrad *et al.*, 2016), making them physiologically sensitive to increased salinities associated with periods of drought and climate change. Suboptimal physiological performance of predators due to environmental stressors may alter predatory behavior (Ferrari *et al.*, 2011) and could potentially decrease predator–prey interactions with native species. Species-specific differences in physiological sensitivity to multiple stressors of increased temperature and salinity projected by climate change may influence long-term distribution and abundance of native fishes in the SFE through both direct and indirect (e.g. species interactions) mechanisms.

The overall goal of this study was to assess the influence of increased temperature and increased salinity as co-occurring stressors on the physiological performance of three SFE fishes: Delta Smelt, Mississippi Silversides, and Largemouth Bass. Here, we determined (1) species-specific tolerance to elevated salinity and temperature, (2) characterized if the sequence in which stressors were experienced (e.g. initial warming, subsequent increased salinity, and vice-versa) affected the upper temperature tolerance (i.e. critical thermal maximum) of fishes and (3) estimated regional differences in the sensitivity of these species to climate change. To determine whether the initial thermal or salinity exposure affected general physiological condition and osmoregulation under a multiple stressor challenge we quantified changes in body condition, hematology, tissue water content and plasma osmolality. We hypothesized native and non-native species would differ in their physiological sensitivity to elevated temperature and salinity. Specifically, we predicted that Delta Smelt would be most severely affected by stressors because of their physiological sensitivity (e.g. lowered tolerance and decreased osmoregulation capacity) and will be most susceptible to extinction under projected climate change. In contrast, we predicted that Mississippi Silversides would be less sensitive to multiple stressors, and Largemouth Bass to be less sensitive to increased temperature but more sensitive to salinity increases than either Silversides or Delta Smelt. Because the SFE is dynamic with respect to temperature, we think specific habitat areas will promote differential geographic vulnerability of each species (determined by relative thermal safety margins). This is important to explore given that specific regions are proposed ‘strongholds’ for Delta Smelt and targets of considerable restoration efforts in the future. Therefore, continued climate change and periodic droughts may favor non-native species persistence in the SFE ecosystem while native fishes continue to decline.

## Materials and methods

### Experimental design

**Figure 2:**
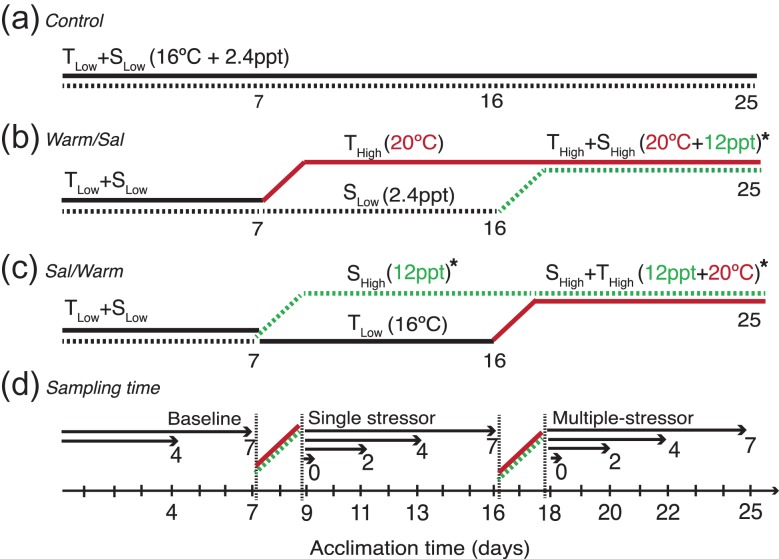
Experimental design, on a scale of days, for initial and subsequent exposures to multiple stressors. Each timeline represents experimental stressor regimes including (**a**) Control conditions, (**b**) Warm/Sal, an initial thermal stressor (T_High_) followed by a subsequent salinity increase (T_High_:S_High_), and (**c**) Sal/Warm, an initial salinity exposure (S_High_) followed by a subsequent temperature increased (S_High_:T_High_). Physiological assessments were made across time (**d**) after each change in exposures to assess responses over time. *Largemouth Bass high salinity exposure was 8 ppt, whereas Delta Smelt and Silversides exposure were 12 ppt.

Delta Smelt, Mississippi Silversides, and Largemouth Bass were exposed to three different experimental stressor regimes over time to test the effect of multiple stressors experienced in series on physiological tolerance. Three experimental regimes (shown in Fig. 2) were as follows: (1) *Control*, where fish were held at constant salinity and temperature conditions for 25 days (T_Low_, S_Low_; 16°C and 2.4 ppt), (2) *Warm/Sal*, where fish spent 7 days at control conditions (T_Low_, S_Low_), followed by 7 days at elevated temperature (T_High_, S_Low_; 20°C and 2.4 ppt), followed by a subsequent salinity increase under elevated temperature conditions for another 7 days (multiple stressor, T_High_, S_High_; 20°C and 12 ppt), and lastly, (3) *Sal/Warm*, where fish were exposed to 7 days to control conditions (T_Low_, S_Low_), followed by increased salinity for 7 days (S_High_, T_Low_; 16°C and 12 ppt), followed by a subsequent increase in temperature under increased salinity conditions for 7 days (same multiple-stressor as in Warm/Sal, S_High_, T_High_; 20°C and 12 ppt). The control temperature of 16°C was selected based on the low-to-middle range of temperatures where Delta Smelt are found in the wild (Bennett, 2005) and for comparison with a previous study (Komoroske *et al.* 2015). The high temperature of 20°C was selected as >90% of wild smelt are caught below 20°C, with most found around 18°C (Bennett, 2005). The experimental salinity exposure of 12 ppt was selected as 92% of smelt are caught below 6 ppt (Komoroske *et al.*, 2016). A pilot experiment exposing four, 7-month old Largemouth Bass (a stricter freshwater species) to 12 ppt for 25 days revealed 50% mortality (data not shown), and hence to ensure that non-lethal physiological and hematological markers could be assessed, the elevated salinity exposure for Largemouth Bass experiments was decreased to 8 ppt. It should be noted that some biologists may argue <22°C and <25°C for Delta Smelt and Largemouth Bass, respectively, may not be considered ‘stressful’. There is uncertainty that temperatures of wild caught Delta Smelt reflect only where biologist choose to sample and may be missing Delta Smelt occupying warmer habitats in the SFE; however, previous studies have demonstrated sub-lethal physiological effects of Delta Smelt at 20°C (Jeffries *et al.*, 2016; Komoroske *et al.*, 2015, 2016). Therefore, for comparative purposes and the uncertainty of Delta Smelt sensitivity to multiple stressors of 20°C and 12 ppt, the described moderate ‘stressors’ were selected acknowledging are not severe.

Each experimental stressor regime was conducted in a separate recirculating system comprised of three replicate tanks to allow for the manipulation of temperature and salinity conditions independently between regimes. Each of the 3 systems had a separate heat pump in which temperature was initially set at 16°C. Salinity treatments of 2.4 ppt were maintained by mixing synthetic sea salt in an external 757 l mixing drum (one for each treatment) plumbed into each system sump. A water change was completed daily by introducing a new 757 l drum of fresh treatment water to the sump. Temperature and salinity increases (Fig. 2) were conducted over a 2-day period (single [Days 7–9] and multiple stressors [Days 16–18]) by increasing temperature from 16 to 20°C at 1°C per 12 h, and increasing salinity from 2.4 to 12 ppt at 2.4 ppt per 12 h by pre-dissolving sea salt in a series of buckets and adding the brine directly to the system sumps. Largemouth Bass salinity treatments were increased 1.4 ppt per 12 h until 8 ppt. Both temperature and salinity changes were homogenous in each replicate tank after 1 h. Temperature, salinity, and dissolved oxygen were measured in each tank and system sump daily using a handheld YSI meter (YSI 85, Yellow Springs, OH, USA) and presented in Table 1. Experimental stressor regimes were conducted on the three species from August to December 2016.

**Table 1:** Average temperature (°C) and salinity (in parts per thousand, ppt) for each experimental stressor regime across acclimation time

		0–7 days (baseline)		9–16 days (single stressor)		18–25 days (multiple stressors)	
Species	Stressor regime	Temperature (°C)	Salinity (ppt)	Temperature (°C)	Salinity (ppt)	Temperature (°C)	Salinity (ppt)
*Delta Smelt*	Control	16.6 ± 0.1	2.4 ± 0.1	16.7 ± 0.1	2.4 ± 0.1	16.7 ± 0.1	2.3 ± 0.1
	Warm/Sal	16.6 ± 0.1	2.4 ± 0.1	20.2 ± 0.3	2.4 ± 0.1	20.4 ± 0.2	12.1 ± 0.1
	Sal/Warm	16.8 ± 0.1	2.4 ± 0.1	16.5 ± 0.5	12.0 ± 0.1	20.5 ± 0.2	12.1 ± 0.1
*Mississippi Silversides*	Control	16.7 ± 0.2	2.3 ± 0.1	16.7 ± 0.1	2.3 ± 0.1	16.9 ± 0.1	2.3 ± 0.1
	Warm/Sal	16.5 ± 0.5	2.4 ± 0.1	20.5 ± 0.3	2.4 ± 0.1	20.6 ± 0.2	12.1 ± 0.1
	Sal/Warm	16.6 ± 0.3	2.4 ± 0.1	15.9 ± 0.1	12.1 ± 0.1	20.8 ± 0.1	12.1 ± 0.1
*Largemouth Bass*	Control	16.1 ± 0.3	2.3 ± 0.1	15.9 ± 0.1	2.3 ± 0.1	16.1 ± 0.3	2.4 ± 0.1
	Warm/Sal	16.0 ± 0.4	2.4 ± 0.1	20.4 ± 0.2	2.5 ± 0.1	20.6 ± 0.1	8.0 ± 0.1
	Sal/Warm	16.2 ± 0.3	2.4 ± 0.1	16.1 ± 0.2	8.0 ± 0.1	20.9 ± 0.1	7.9 ± 0.1

The Control represents with Low Temperature (T_Low_) and Low Salinity (S_Low_) across time, whereas Warm/Sal and Sal/Warm regimes have an initial exposure to either High Temperature (T_High_) or High Salinity (S_High_), and both are followed by the same multiple stressor exposure (T_High_ and S_High_). Each value is the average (±SD) for each exposure period including the baseline week (Days 0–7), the initial single stressor week (Days 9–16) and the subsequent multiple stressor week (Days 18–25).

### Fish culture and maintenance

Juvenile Mississippi Silversides (*Menidia beryllina*) were obtained from Aquatic BioSystems (Fort Collins, CO, USA) in July 2016 at 135 days post hatch (dph). Silversides were immediately separated into nine 400 l tanks upon arrival (*n* = 90–96/tank), acclimated to 16°C and 2.4 ppt for 2 weeks before the experimental regimes (*n* = 3 tank replicates/regime) began at ~150 dph. Juvenile Delta Smelt (*Hypomesus transpacificus*) were spawned and reared at the University of California Davis—Fish Culture and Conservation Laboratory (FCCL) in Byron, CA using optimal culture conditions (16°C, 0.4 ppt [Lindberg *et al.*, 2013]). Smelt were transported in September 2016 at ~135 dph, separated into nine 400 l tanks (*n* = 90–99/tank with 3 tank replicates/stressor regime), and acclimated to 16°C and 2.4 ppt for one week until the experiment began at ~145 dph. Silversides and Smelt were fed 3% body weight (g) per day with a mix of Hikari Plankton (25%; semi-float), and BioVita starter (50% of #0 crumble and 25% #1 crumble). Largemouth Bass (*Micropterus salmoides*) juveniles were transported from a commercial aquaculture facility (The Fishery Inc., Galt, CA, USA) at ~7 months of age in late October 2016. Bass were immediately split into nine 682 l tanks (*n* = 60–75 per tank with 3 tank replicates per regime) upon arrival, and acclimated at 16°C and 2.4 ppt for 4 weeks until the experiment began. Bass were fed a pellet diet (Skretting, Tooele, UT, USA), twice daily, at 1% body weight per day. Feeding rations for each species remained the same from acclimation through experimental stressor testing.

### Whole-organism physiological tolerance

#### Upper thermal tolerance

We determined upper temperature tolerance in each species of fish using critical thermal maximum methodology (CTMax: Beitinger *et al.*, 2000; Beitinger & Bennett, 2000). CTMax trials were conducted on 12 fish from each experimental stressor regime (*n* = 4 per tank replicate) during the baseline week (Days 4, 7), the initial stressor week (Days 9, 11, 13, 16) and the subsequent multiple stressor week (Days 18, 20, 22, 25). Each species was given 30 min in individual CTMax chambers with their respective experimental regime water conditions before the CTMax trial began. Water temperature was raised 0.3°C min^–1^ until fishes exhibited loss of equilibrium (LOE), a common CTMax endpoint used to determine ecological upper thermal tolerance (Beitinger *et al.*, 2000; Beitinger & Bennett, 2000; Komoroske *et al.*, 2014; Jeffries *et al.*, 2016). Once fish demonstrated LOE, temperature was recorded with a calibrated immersion thermometer (to 0.1°C), and fish were immediately removed from the chamber and placed in individually labeled recovery tanks (9.5 l or 18.9 l) at the fish’s specific regime conditions (i.e. if fish came from a 12 ppt or 20°C exposure, they recovered in those conditions). Only fish that survived 24 h following the CTMax trial were included in the dataset. Smelt and Silversides were measured in 1.5 l chambers that were painted black to reduce potential visual stress. Chambers were then placed in a 115 l water-bath at the appropriate acclimation temperature (16 or 20°C) and temperatures were ramped using two 800-Watt submersible heaters and water pumps for even heating of water. Six consecutive CTMax trials with 6 chambers each were conducted on each day for Silversides and Delta Smelt. Largemouth Bass were measured individually in 18 l aquarium tanks affixed with an acrylic lid, 4 of these tanks were placed into two larger water baths for 8 individuals per trial and 5 total CTMax trials on each day. Each of these water baths contained an 1800 W heater, two 500 W heaters and had two water pumps for even heating and circulation of water. Treatments and tank replicates were randomized throughout the day for all species. All CTMax chambers contained an air-stone to ensure that O_2_ levels did not decrease during the trial.

#### Fish sampling

On Days 4, 7 (baseline), 9, 11, 13, 16 (single stressor), 18, 20, 22 and 25 (multiple stressors), fishes (*n* = 9 per time point) were rapidly euthanized in an overdose of tricaine methanesulfonate (50 mg L^–1^ MS-222), and standard length and mass were recorded. Fishes were then immediately sampled for blood and muscle tissue (near the caudal peduncle) for later sub-organismal physiological assessments of sensitivity. Body condition factor (K) was calculated using Fulton’s condition factor as:

$$K\;=\;100\;\times \;\frac{W}{L^{3}}$$

where *W* is the wet mass of the fish in grams, and *L* is the standard length (tip of snout to caudal peduncle) in cm. Body condition factor was used to determine if experimental stressor regimes decreased health condition across acclimation time (Komoroske *et al.*, 2015).

### Sub-organismal physiological sensitivity

Hematocrit (% red blood cells/total volume) was measured to determine if hematological alterations occurred to support increased blood oxygen carrying capacity. Blood was collected from the caudal vasculature of euthanized fish with a heparinized micro-hematocrit capillary tube (60 mm calibrated tip, 0.5 mm inner diameter [ID] for Smelt and Silversides and 70 mm calibrated tip, 1 mm ID for Bass). Tubes were rapidly sealed with a putty compound and spun on a micro-hematocrit centrifuge for 3 min to separate the red blood cells from the plasma. Percent hematocrit was read in duplicate and recorded.

Plasma was collected by scoring the hematocrit tube with a file, separating the plasma from the red blood cells. Plasma was then pipetted into a micro-centrifuge tube, frozen on dry ice and stored at –80°C until analysis. Plasma osmolality (mOsm kg^–1^) was quantified to assess osmotic imbalances that might have occurred in response to thermal and osmotic experimental stressor regimes. For Delta Smelt and Mississippi Silversides osmolality was measured in 2 μl plasma samples using a vapor pressure osmometer (Vapro 5600, Wescor Biomedical Systems, Logan, UT, USA). Due to the small amount of blood and plasma collected for Smelt and Silversides, when possible individual samples were analyzed. If individual volume was insufficient, samples were pooled for the respective replicate tank (Komoroske *et al.*, 2016). Largemouth Bass osmolality was analyzed in triplicate using 10 μl of plasma. Due to size-limited plasma samples, not all acclimation days were analyzed. Osmolality was measured after 7 days (baseline), 9 and 16 (0 and 7 days after the initial exposure), and 18 and 25 days (0 and 7 days following the subsequent multiple stressor) to evaluate acute and short-term osmoregulatory changes.

Alterations in muscle water content can indicate if osmoregulation abilities of fishes have been compromised. A cross-section of muscle tissue, sampled at the caudal peduncle, was used to quantify muscle water content. Muscle water content was measured as percentage tissue water and was calculated as:

$$\%\;tissue\;water\;=\;\frac{WM-DM}{WM}\;\times \;100$$

where *WM* is initial wet mass recorded after sampling the tissue, and *DM* is the dry mass of the tissue following tissue drying for 24 h at 60°C (Sullivan and Somero, 1980; Wilkie *et al.*, 2015).

### Thermal safety margins

To provide an ecological link between upper temperature tolerances of fish species under the different experimental stressor regimes to temperatures experienced in nature, thermal safety margins (TSM) were calculated (Deutsch *et al.*, 2008). TSM, the difference between habitat temperatures and upper thermal limits (CTMax) provides an index of how close fishes are currently living to their limits and projects potential vulnerability to climate change increases in water temperature (e.g. Brown *et al.*, 2016). TSMs of fishes acclimated to 20°C (an indication of acclimation capacity to warming) were calculated as:

$$TSM\;=\;CTMax_{20{}^{\circ}C\;acclimated}-\;T_{Habitat}$$

TSMs were calculated for a habitat temperature of 20°C, simulating the current study’s experimental temperature stressor regime. A habitat temperature of 20°C is also close to the mean temperature of 18.9°C where many early life stages of Delta Smelt were historically caught in field surveys in summer and fall, seasons when temperature stress is most likely to occur (Jeffries *et al.*, 2016). Because different regions within the SFE have different maximum habitat temperatures and average daily temperatures from July to August, TSMs were also calculated for three other regions within the SFE (Fig. 1): (1) the West and North Delta regions (maximum 25°C, average range 21–23°C), (2) Suisun and San Pablo Bays (maximum 24°C, average range 19–21°C) and (3) the South Delta (maximum 28°C, average range 23–26°C) as summarized recently (Interagency Ecological Program MAST, 2015). Increased water temperature (estimated 2–4°C) is one of the major changes predicted for the SFE (Cloern *et al.*, 2011; Wagner *et al.*, 2011), and current high temperatures in the South Delta region may become estuarine-wide by the year 2100. Therefore, South Delta TSMs provide a good estimate of what TSMs may be across all habitat regions with warming.

#### Statistical analyses

Statistical analyses were performed using R (v3.1.3; R Development Core Team, 2013) with associated packages, *car*, *lme* (Pinheiro *et al.*, 2017) and *lsmeans* (Lenth, 2016), and an alpha value set at 0.05. All datasets were first visually inspected for assumptions of normality and homogeneity of variances using figures of Q-Q plots and residuals vs. fitted values and frequency, as well as residuals versus fixed factors of species, experimental stressor regimes, acclimation time, and replicate tanks. Data were log transformed when needed and analyzed using linear regressions (LR) and linear models (LMs). Model summary tables were generated using *Anova* function, and post-hoc Tukey comparisons were conducted using the *lsmeans* to determine differential responses by stressor regime across acclimation time, as well as between each regime at a given time. LMs were conducted for each species separately since Largemouth Bass had different salinity exposure values (8 ppt) compared to Delta Smelt and Silversides (12 ppt). Since each sampling time point was an independent subset of fish, LRs were conducted to assess the impacts of stressor regimes and acclimation time on several related growth metrics including wet mass, standard length and calculated body condition factor. A univariate LM was conducted for CTMax with stressor regime and acclimation time as predictors and fish size as a covariate in the model. Since the elevated temperature exposure was comparable across all species for the initial stressor period (Warm/Sal), a LM was conducted to determine if CTMax under control conditions (Day 7) and after a week of increased temperature (Day 16) differed by species. A separate univariate LM was conducted for each sub-organismal metric (hematocrit, % tissue water, and osmolality [for LMB only]) due to missing values and uneven sample sizes. Only fish sampled for physiological metrics were included in the body condition factor measures (including wet mass and length) as CTMax procedures may have altered mass and body condition.

Lastly, species differences in ecological thermal safety margins (TSM) were tested with an LM with species and SFE region as fixed factors and the calculated TSM. TSM values included in the model were pooled from experimental Days 11, 13 and 16 from species acclimated to 20°C since CTMax were not statistically different, *P* > 0.05. All fish were originally nested within each replicate tank in each LM; however, with no significant effects of tank or interaction with treatment regimes, replicate tank was removed from the model.

## Results

### Whole organism physiological tolerance

#### Upper thermal tolerance

**Figure 3:**
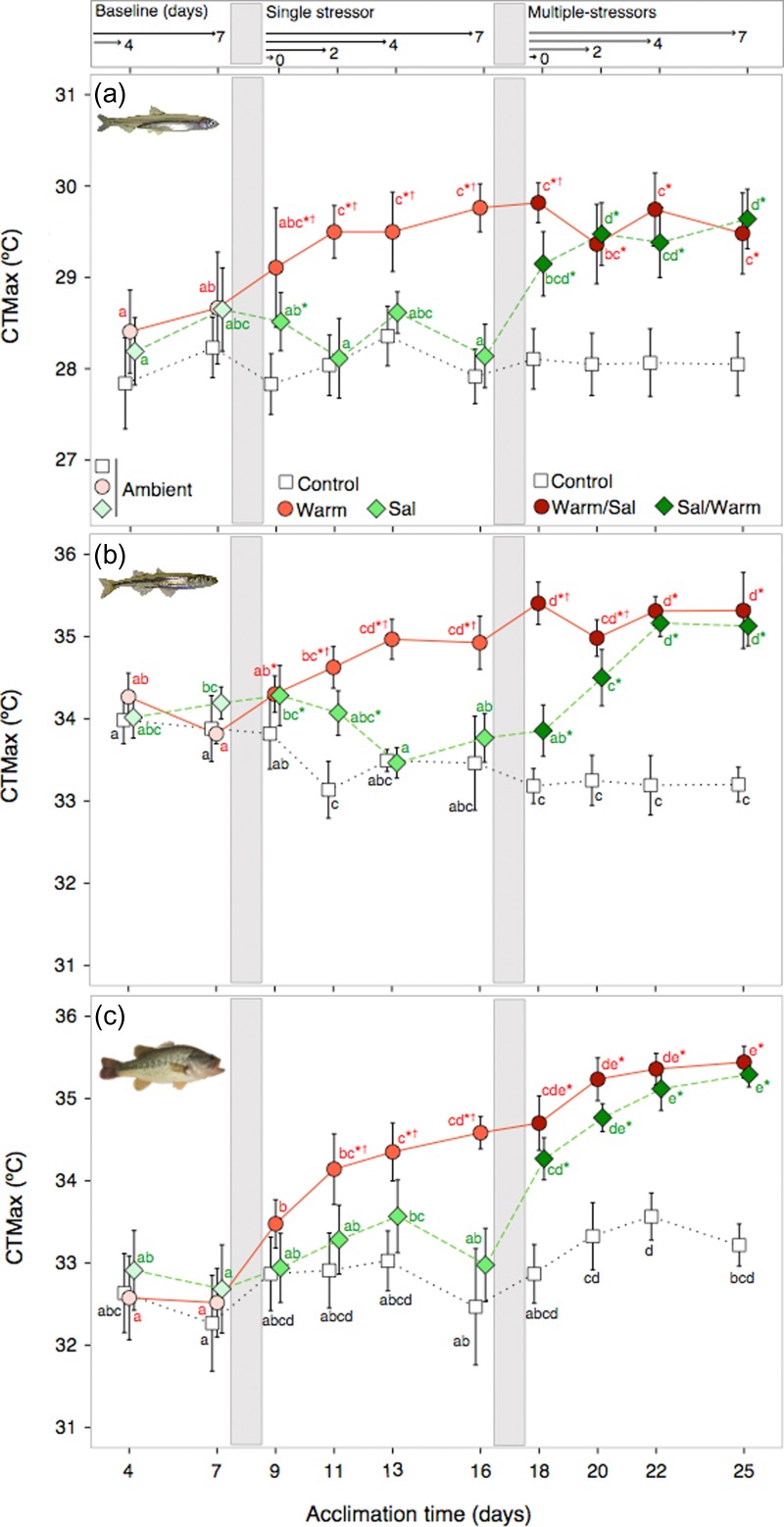
Critical thermal maximum (CTMax) ± 95% confidence (CI) of ‘San Francisco Estuary species’. species. Within each panel, (**a**) Delta Smelt, (**b**) Mississippi Silversides and (**c**) Largemouth Bass average CTMax is given for *n* = 10–12 individuals per point. Note the *y*-axis of Delta Smelt differs from the other species. Vertical gray bars indicate exposure changes from the baseline to initial single stressors and then to subsequent multiple-stressor periods. Letters indicate significant differences (*P* < 0.05) in CTMax within a given stressor regime across acclimation time (Control [white], Warm/Sal [red], and Sal/Warm [green]). Asterisks indicate a difference from the control at each day, and daggers represent a difference between Warm/Sal and Sal/Warm stressor regimes at each day (Tukey, *P* < 0.05).

Each species showed similar responses in upper thermal tolerance to experimental stressor regimes across acclimation time such that elevated temperature increased CTMax and elevated salinity had little to no effect, depending on species (Fig. 3). CTMax of Delta Smelt was significantly affected by stressor regimes (*F*_23,21_ = 107.98, *P* < 0.001); however, the effect of stressor regime was dependent on acclimation time (*F*_9,321_ = 8.69, *P* < 0.001) indicated by a significant interaction (*F*_9,321_ = 4.84, *P* < 0.001) between the two factors. There was no effect of fish size (length, *F*_1,321_ = 1.35, *P* = 0.25) on CTMax of Delta Smelt within the length ranging from 25.3 to 54.5 mm. Acclimation to elevated temperature increased CTMax (see Days 9–16 in Warm/Sal, and 18–25 in Sal/Warm), but salinity had no effect on CTMax (see Tukey results in Fig. 3a). Mississippi Silverside CTMax showed a significant interaction (*F*_18,309_ = 15.12, *P* < 0.001) between the main effects of stressor regime (*F*_2,309_ = 253.42, *P* < 0.001) and acclimation time (*F*_9,309_ = 7.02, *P* < 0.001), as well as an effect of fish size (standard length, *F*_1,309_ = 4.64, *P* = 0.03). Larger Silversides had slightly higher CTMax compared to smaller Silversides. Post-hoc Tukey results indicated temperature not salinity was responsible for the increased CTMax (see values in Fig. 3b). After 7 days of exposure to an initial increase in temperature (Warm/Sal), CTMax of Silversides increased by ~1.5°C (Tukey, *P* < 0.05), and remained stable under the subsequent salinity exposure (*P* > 0.05). The initial salinity exposure (Sal/Warm) had no effect on Silverside CTMax; however, with the subsequent exposure to increased temperature CTMax increased to match the peak CTMax of fish exposed to increased temperature in the Warm/Sal regime. Largemouth Bass CTMax was also significantly affected by stressor regimes (*F*_2,320_ = 141.50, *P* < 0.001), and acclimation time (*F*_9,320_ = 54.34, *P* < 0.001), with a significant interaction (*F*_18,320_ = 7.52, *P* < 0.001) between regime and time. There was with no effect of size of Largemouth Bass on CTMax (length, *F*_1,320_ = 0.002, *P* = 0.96, Fig. 3c). In contrast to Smelt and Silversides, increased salinity and temperature interacted in an additive fashion to increase CTMax in Largemouth Bass, such that after the multiple stressor exposure the increase in CTMax was a sum of the initial (after Day 16) and subsequent temperature and salinity exposures (after Day 25) (~2.6°C total, Fig. 3c).

**Figure 4:**
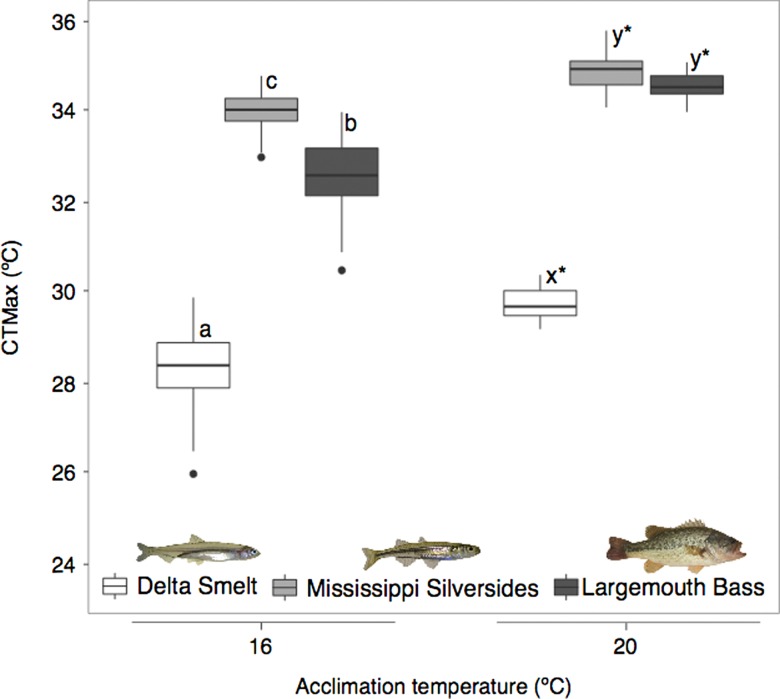
Species comparisons of upper tolerance (CTMax) and acclimation capacity. Boxplots are colored by species: Delta Smelt (white), Silversides (gray), and Largemouth Bass (dark gray). For boxplots, the center line represents the median, the box represents the inter-quartile range (IQR), the whiskers extend 1.5 times IQR, and black points represent values outside 1.5 the IQR. Letters indicate differences between species within each temperature exposure (*P* < 0.05), and asterisks represent a significant increase in thermal tolerance within each species after 20°C acclimation (*P* < 0.05).

Comparative analyses of CTMax of species under reference control conditions and 7 days exposure to warming (Warm/Sal) showed CTMax at a given acclimation temperature and capacity to acclimate differed by species (Fig. 4; *F*_2,135_ = 932.52, *P* < 0.0001), temperature (*F*_1,135_ = 136.63, *P* < 0.0001), with a significant interaction between species and temperature (*F*_2,135_ = 7.82, *P* = 0.001). Under baseline control conditions (16°C, 2,4 ppt, Day 7) CTMax was different between each species with both non-native species having higher upper temperature tolerances than native Delta Smelt (Tukey, *P* < 0.05). Silverside and Largemouth Bass CTMax were 5.4°C and 3.9°C higher, respectively, than Delta Smelt at 16°C (*P* < 0.05). After a 1-week exposure to 20°C, CTMax in all species was significantly increased (*P* < 0.05). The largest increase in CTMax was observed in Largemouth Bass (+2.1°C), such that CTMax of fish increased to almost that of Silversides at 20°C (only 0.4°C difference, *P* > 0.05).

#### Body condition factor

Body condition factor of Delta Smelt acclimated to stressor regimes (Warm/Sal and Sal/Warm) across acclimation time was similar to control fish (*P* > 0.05, see linear regression analyses in Table S1). Growth metrics including length and mass of Delta Smelt also were unaffected by stressor regimes across the 25 day exposures (*P* > 0.05, Table S1; Table 2). Body condition factor of Mississippi Silversides was also unaltered by stressor regimes (*P* > 0.05); however, in contrast, both standard length (*T *= 2.03, *P* = 0.043) and mass (*T* = 2.048, *P* = 0.042) of Silversides in Warm/Sal were greater than control fish across acclimation time (see Table S1 for model results, Table 2 for mean ± SE values). Largemouth Bass body condition factor, length and mass across acclimation time were similar for all experimental stressor regimes and the control (*P* > 0.05, Table S1; Table 2).

**Table 2:** Mean (±SD) of physiological and hematological markers of San Francisco Estuary fishes.

		Day 7 (baseline)			Day 16 (single stressor)			Day 25 (multiple stressors)		
Species & Physiological Biomarker	*n*	Control	Warm/Sal	Sal/Warm	Control	Warm/Sal	Sal/Warm	Control	Warm/Sal	Sal/Warm
*Delta Smelt*										
Mass (mg)	19–21	357 ± 86	408 ± 111	415 ± 133	317 ± 156	478 ± 221	374 ± 120	441 ± 293	579 ± 202	489 ± 202
Standard length (mm)	19–21	36.3 ± 2.4	38.8 ± 2.8	38.5 ± 3.2	34.8 ± 4.8	39.7 ± 5.8	37.3 ± 3.6	37.8 ± 5.9	41.8 ± 4.2	39.4 ± 4.9
Body condition factor	19–21	0.7 ± 0.1	0.7 ± 0.1	0.7 ± 0.1	0.7 ± 0.1	0.7 ± 0.1	0.7 ± 0.1	0.7 ± 0.1	0.8 ± 0.1	0.8 ± 0.1
Hematocrit	3–9	46 ± 10	44 ± 8	42 ± 5	43 ± 6	44 ± 5	43 ± 8	42 ± 5	44 ± 11	41 ± 9
% tissue water	7–9	78.5 ± 1.1	78.7 ± 0.7	78.3 ± 0.4	78.3 ± 0.9	77.6 ± 1.6	77.4 ± 0.4	80.4 ± 1.4	79.0 ± 0.3	80.4 ± 0.7
Osmolality (mOsm kg^–1^)^a^	1–6	371 ± 29	370 ± 7	358 ± 3	372 ± 19	351 ± 16	381 ± na	367 ± 20	368 ± 16	368 ± 15
*Mississippi Silverside*										
Mass (mg)	17–21	379 ± 136	355 ± 109	398 ± 127	401 ± 115	448 ± 145	421 ± 123	430 ± 108	454 ± 117	447 ± 144
Standard length (mm)	17–21	32.6 ± 3.8	32.0 ± 2.7	33.5 ± 3.6	33.3 ± 3.0	34.0 ± 3.3	33.8 ± 3.5	34.7 ± 2.6	35.2 ± 2.7	35.0 ± 3.2
Body condition factor	17–21	1.1 ± 0.1	1.1 ± 0.1	1.0 ± 0.1	1.1 ± 0.1	1.1 ± 0.1	1.1 ± 0.1	1.0 ± 0.1	1.0 ± 0.1	1.0 ± 0.6
Hematocrit	5–9	44 ± 9	40 ± 6	49 ± 8	43 ± 8	41 ± 5	48 ± 12	42 ± 7	45 ± 7	42 ± 9
% tissue water	6–12	75.8 ± 1.8	74.9 ± 1.4	75.6 ± 0.8	75.7 ± 1.3	75.2 ± 1.4	75.3 ± 0.8	74.5 ± 1.3	75.0 ± 0.9	74.1 ± 0.9
Osmolality (mOsm kg^–1^)^a^	1–6	372 ± 15	392 ± na	383 ± 11	–	–	–	394 ± 4	390 ± 6	385 ± 12
*Largemouth Bass*										
Mass (g)	19–21	197 ± 42	164 ± 48	182 ± 43	198 ± 49	207 ± 35	203 ± 48	234 ± 42	214 ± 45	196 ± 45
Standard length (cm)	19–21	22.3 ± 1.9	21.3 ± 1.9	21.9 ± 1.8	22.5 ± 2.3	22.5 ± 1.8	22.1 ± 2.5	22.9 ± 2.3	22.2 ± 2.1	22.0 ± 2.2
Body condition factor	19–21	1.8 ± 0.4	1.7 ± 0.3	1.7 ± 0.5	1.7 ± 0.3	1.9 ± 0.4	1.9 ± 0.4	2.0 ± 0.4	2.0 ± 0.5	1.9 ± 0.5
Hematocrit	7–9	42 ± 5	42 ± 7	41 ± 5	35 ± 4	36 ± 4	36 ± 4	39 ± 6	39 ± 6	34 ± 5
% tissue water	8–9	74.2 ± 1.4	74.7 ± 1.8	73.5 ± 1.5	74.5 ± 1.8	73.4 ± 1.4	74.0 ± 1.1	73.7 ± 1.08	74.0 ± 2.0	74.0 ± 0.8
Osmolality (mOsm kg^–1^)	6–9	291 ± 13	295 ± 14	301 ± 8	302 ± 12	306 ± 6	313 ± 10	296 ± 7	316 ± 11	312 ± 13

Values are given for each experimental stressor regime. Control (T_Low_:S_Low_), Warm/Sal [T_High_:S_Low_ → T_High_:S_High_], and Sal/Warm [T_Low_:S_High_ → T_High_:S_High_] are provided only after 7 days acclimation in each stressor regime (i.e. 7, 16 and 25 days).

^a^Due to small sample sizes these parameters were not statistically tested but average values are presented when possible.

### Sub-organismal physiological sensitivity

Mean values of hematocrit, osmolality and muscle tissue water content across each exposure period are given in Table 2 for Delta Smelt, Mississippi Silversides, and Largemouth Bass. Delta Smelt hematocrit was altered by stressor regime (F_2,183_ = 4.487, *P* = 0.012), independent of acclimation time (*F*_9,183_ = 1.77, *P* = 0.076) with no interaction between regime and time (*F*_18,183_ = 0.758, *P* = 0.747). Overall, Delta Smelt in Warm/Sal had greater hematocrit at 44 ± 1 (mean ± SE across all time points; lsmeans Tukey, *P* < 0.05) compared to 40 ± 1 in the control or 42 ± 1 in Sal/Warm. Tissue water content was altered by stressor regime (*F*_2,183_ = 4.719, *P* = 0.001) and acclimation time (*F*_9,183_ = 6.367, *P* < 0.001) with no interaction between regime and time (*F*_18,183_ = 0.680, *P* = 0.829). Overall, tissue water content (mean ± SE across time points) of Delta Smelt in Warm/Sal was 0.7 ± 0.2% lower than control fish (lsmeans Tukey, *P* < 0.05), but tissue water content of fish in Sal/Warm was similar to control fish (*P* > 0.05). In general, tissue water content of Delta Smelt did increase over acclimation time from 78.5 ± 0.3 (mean ± SE across all stressor regimes) at 7 days to 79.9 ± 0.3 after 25 days.

Hematocrit of Silversides was unaffected by stressor regime (*F*_2,187_ = 0.636, *P* = 0.530), and acclimation time (*F*_9,187_ = 0.216, *P* = 0.991) with no interactions between the factors (*F*_18,187_ = 0.695, *P* = 0.814). Average hematocrit was 43 ± 3 (mean ± SE across all stressor regimes and time points). Tissue water content in Silversides varied by acclimation time (*F*_9,218_ = 11.94, *P* < 0.001), with no effect of stressor regime (*F*_2,218_ = 0.396, *P* = 0.673) and no interactions (*F*_18,218_ = 1.492, *P* = 0.095). Overall tissue water content ranged from 74.4 ± 0.3% to 75.9 ± 0.3% from Days 4 to 25; however, an unusual spike to 78.2 ± 0.3% in tissue water content was measured at 13 days that was significantly different from all other days (lsmeans Tukey, *P* < 0.05).

**Figure 5:**
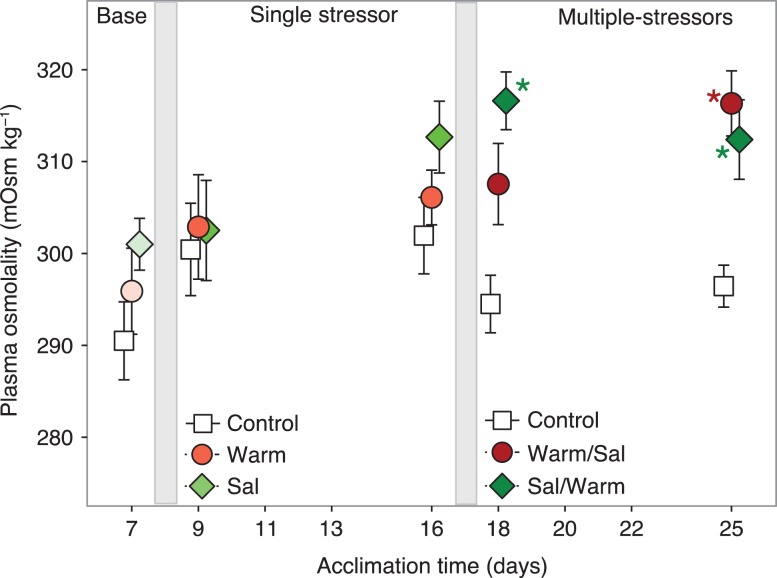
Largemouth Bass plasma osmolality across experimental stressor regimes. Each point represents the mean (±SE) of *n* = 9 for each acclimation time point and stressor regime. Asterisks indicate a difference between stressor Warm/Sal [T_High_ → T_High_:S_High_, red] or Sal/Warm [S_High_ → T_High_:S_High_, green] from the Control ([T_Low_:S_Low_, white] within each day (lsmeans Tukey, *P* < 0.05). At 7 days (Base) all experimental regimes were at T_Low_:S_Low_.

Largemouth Bass hematocrit was significantly altered by stressor regime (*F*_2,223_ = 5.030, *P* = 0.007) and acclimation time (*F*_9,223_ = 4.929, *P* < 0.001), with no interaction (*F*_18,223_ = 1.160, *P* = 0.296). Overall, Bass in Sal/Warm had the lowest hematocrit at 37 ± 1 (mean ± SE across all time points) compared to 40 ± 1 of control fish (lsmeans Tukey *P* < 0.05) or 39 ± 1 in Warm/Sal. Tissue water content in Bass muscle was not altered by stressor regime (*F*_2,239_ = 0.083, *P* = 0.920) or acclimation time (*F*_9,239_ = 1.330, *P* = 0.222) with no interactions between factors (*F*_18,239_ = 1.026, *P* = 0.4317). Plasma osmolality (Fig. 5, measured the first and last day within each exposure period) significantly increased by stressor regime (*F*_2,111_ = 11.223, *P* < 0.001) and acclimation time (*F*_4,111_ = 4.778, *P* = 0.001), with no interactions (*F*_8,111_ = 1.305, *P* = 0.249).

### Thermal safety margins

The TSMs calculated to assess the window for buffering between upper temperature tolerance (CTMax) and maximum habitat temperatures significantly differed by species (*F*_2,400_ = 1157, *P* < 0.001) and the SFE region where the TSM was calculated (*F*_3,400_ = 1096, *P* < 0.001). Both non-native species had larger TSMs (ranging from 6.1 to 14.1°C [Silversides] and 5.3 to 13.3°C [Largemouth Bass]) than native Delta Smelt (ranging from 0.6 to 8.6°C, Tukey, *P* < 0.05, Table 3).

**Table 3:** Estimated thermal safety margins (TSM) of native and non-native species for given San Francisco Estuary (SFE) habitats (see Fig. 1 for general region locations)

Species	SFE Habitat	TSM (°C)	SE	lower.CL	upper.CL
*Delta Smelt* (*n* = 33)					
	20°C exposure	8.6	0.18	8.3	8.9
	West/North Delta	3.6	0.18	3.3	3.9
	Suisun & San Pablo Bays	4.6	0.18	4.3	4.9
	South Delta	0.6	0.18	0.3	0.9
*Mississippi Silverside* (*n* = 34)					
	20°C exposure	14.1	0.17	13.7	14.4
	West/North Delta	9.1	0.17	8.7	9.4
	Suisun & San Pablo Bays	10.1	0.17	9.7	10.4
	South Delta	6.1	0.17	5.7	6.4
*Largemouth Bass* (*n* = 36)					
	20°C exposure	13.3	0.17	13.0	13.7
	West/North Delta	8.3	0.17	8.0	8.7
	Suisun & San Pablo Bays	9.3	0.17	9.0	9.7
	South Delta	5.3	0.17	5.0	5.7

Average thermal margins (CTMax_20°C acclimated fish_ – T_habitat_) are given with SE as the standard error, and the lower and upper confidence levels (CL).

## Discussion

This study highlights comparative physiological responses of native and non-native fish species to multiple stressors resulting from predicted climate change and associated weather extremes, particularly drought and extreme heat in an estuary. Specifically, this study provides evidence that an initial sub-lethal stressor of elevated temperature or elevated salinity does not compromise the ability to cope with the addition of a secondary heterologous stressor (multiple stressor scenario) in three species of the San Francisco Estuary (SFE). Here, both native and non-native species increased their upper thermal tolerance following acclimation to high temperature, independent of elevations in salinity. In contrast, exposure to elevated salinity increased upper thermal tolerance only in Largemouth Bass. Thermal safety margins of 20°C-acclimated fish were greatest in non-native species compared to native Delta Smelt, and indicated that Smelt are a species currently occupying habitats approaching their upper temperature tolerance limits.

### Plasticity in upper thermal tolerance

All species were able to acquire additional upper temperature tolerance with 7 days of exposure to +4°C above control temperature (Fig. 3); however, species-specific plasticity in thermal tolerance was evident. Delta Smelt increased CTMax by ~1.2°C, whereas non-native species increased thermal tolerance by ~1.5 to 2°C (Fig. 3). Plasticity in upper thermal tolerance is common in fishes and known to be modulated by warm acclimation, including for Delta Smelt (Komoroske *et al.*, 2014) and Largemouth Bass (see review Beitinger *et al.*, 2000; Beitinger & Bennett, 2000). Based on previous findings, Largemouth Bass have been shown to acquire greater upper temperature tolerance if acclimated to temperatures above 20°C (Currie *et al.*, 1998). In contrast to warming, exposure to increased salinity did not increase upper temperature tolerance limits in prey species (Delta Smelt and Silversides, Fig. 3a,b). Similarly, acute salinity exposures of 2, 6, 18 and 34 ppt have previously been shown to have no effect on CTMax in adult Delta Smelt (Komoroske *et al.*, 2014). Largemouth Bass, were differentially affected by increased temperature and salinity compared to Silversides and Delta Smelt. Bass CTMax was increased by exposure to increased salinity, but it is of note that Bass were tested at 8 ppt and not 12 ppt as the prey species. It is unclear if the two-step increase in CTMax of Largemouth Bass (seen in Warm/Sal and Sal/Warm regimes) was the result of an interactive additive effect of elevated temperature and salinity (e.g. sum of single effects of salinity and temperature), or alternatively, if Bass’ CTMax increased in the multiple-stressor period because the fish were still acclimating to warm temperatures, independent of salinity (Fig. 3c). Overall, the sequential elevation in temperature and salinity (or salinity followed by temperature) did not negatively compromise upper thermal tolerance in any of the SFE species tested. The rate of acquired temperature tolerance varied among species. For example, Delta Smelt and Silversides rapidly reached a new baseline for upper thermal tolerance 2–4 days following the elevated temperature exposure, whereas Bass upper temperature tolerance had not stabilized after 7 days, demonstrating that acclimation to warming takes longer in Largemouth Bass than the prey species in this study (Fig. 3).

Although Delta Smelt and Silversides had faster rates of acquiring upper thermal tolerance, it is possible that both species could have acquired additional upper thermal tolerance if acclimation temperatures were higher (like Largemouth Bass, Currie *et al.*, 1998). No other studies have assessed the capacity of Delta Smelt to acquire thermal tolerance when acclimated to temperatures above 20°C. Juvenile Delta Smelt acclimated to a continuous increase in temperature at 1°C per day showed a chronic lethal maximum temperature (i.e. the temperature at which 50% mortality of fish is observed) of 27–28°C (Komoroske *et al.*, 2014). Additional studies should test the maximum acclimation potential of Delta Smelt. For example, if smelt were acclimated to 22 or 24°C for 2 weeks could they further increase their upper thermal limits or have they in fact reached their ultimate thermal limit. It is of note that the present study recorded some of the highest acute upper temperature tolerance limits for juvenile Delta Smelt after warm acclimation with a mean CTMax of 29.7°C ± 0.2 (mean ± SE). Twenty-five percent (36 individuals of 144) of Delta Smelt actually reached 30.0–30.7°C until sudden loss of equilibrium. Together, our findings suggest that juvenile Delta Smelt may indeed have more plasticity in upper tolerance limits than previously described (Swanson *et al.*, 2000; Komoroske *et al.*, 2014, Jeffries *et al.*, 2016).

### Sensitivity to elevated temperature and salinity

Indices of sub-organismal performance of Delta Smelt were sensitive to warming and elevated salinity regimes in the current study. Delta Smelt showed physiological sensitivity to the Warm/Sal regime in particular, as evident by increased hematocrit and decreased muscle tissue water content compared to the control fish (Table 2). Elevated hematocrit (i.e. an increase in red blood cells) after 2 weeks of warming indicates additional oxygen carrying capacity may have been needed to support elevated metabolic demands of warming and/or increase in salinity. In a previous study, when exposed to 20°C, Delta Smelt larvae responded by elevating aerobic metabolism and upregulating heat stress repair genes (Jeffries *et al.*, 2016), while adults had a reduced capacity to regulate cellular repair mechanisms under warming (Komoroske *et al.*, 2015). While it is unclear if changes in hematocrit and muscle tissue water content were in response to warming or the subsequent salinity stressor, previous studies have shown Delta Smelt are relatively insensitive to salinity increases to 12 ppt (Komoroske *et al.*, 2014; Kammerer *et al.*, 2016; Hammock *et al.*, 2017). Given the relative insensitivity to elevated salinity, the elevated hematocrit levels in juvenile Delta Smelt in Warm/Sal were likely a heightened response to warming and not salinity. Lastly, Delta Smelt showed stable body condition following the series of exposures to elevated temperature and salinity after 25 days, similar to previous studies in juveniles and adults (Komoroske *et al.*, 2014; Kammerer *et al.*, 2016). Delta Smelt in this study were likely able to maintain energy allocation required for maintenance mechanisms and body growth when fed to satiation. Under natural conditions, if food resources are low, the physiological adjustments required to maintain homeostasis under warming and changes in salinity may be energy limited. Although all species irrespective of origin (native versus non-native) require sufficient resources to maintain homeostatic mechanisms, species with greater thermal sensitivity and lower tolerance, such as Delta Smelt, may be more susceptible to a mismatch in the energy supply required to meet increased energetic demands at lower temperatures in a low food environment compared to eurythermal non-native species.

Physiological sensitivity of Mississippi Silversides was not affected by multiple stressors of warming and increased salinity, whereas Largemouth Bass had altered physiology indicating sensitivity to stressor regimes. Silversides have been described previously as extremely eurythermal (10–38°C, Beitinger *et al.*, 2000; Interagency Ecological Program *et al.*, 2018a,b) and euryhaline (0–35 ppt, Mahardja *et al.*, 2016). Our findings of unaltered hematocrit and muscle tissue water content, as well as increased growth suggest elevated salinity and warming may enhance physiological optima of Mississippi Silversides. Similarly, growth capacity of Atlantic Silversides (*Menidia menidia*) has been shown to increase with warming (Baumann and Conover, 2011). In contrast, Largemouth Bass in the present study did not experience increased growth from warming and/or elevated salinity (potentially due to a lower feeding rate, Niimi and Beamish, 1974), and alterations in hematocrit and plasma osmolality were evident following the multiple stressors. Hematocrit decreased in the multiple stressor regime with the initial salinity increase (Sal/Warm), whereas plasma osmolality increased in both serial stressor regimes of high temperature and salinity by the end of the experiment (Day 25, Fig. 5), suggesting some degree of osmoregulatory imbalance (Meador and Kelso, 1990). Largemouth Bass osmolality in this present study was 291–301 mOsm kg^–1^ (baseline), which is roughly isosmotic to 8 ppt. Many invasive species in the SFE have been shown to predate on Delta Smelt (although in low numbers, likely due to the rarity of the species; Schreier *et al.*, 2016); however, if Delta Smelt have greater performance in higher salinities, the threat of predation may be reduced due to species-specific differences in osmotic tolerance, at least for Largemouth Bass predators. More research investigating osmoregulation mechanisms of non-native species, particularly predators in the SFE, is warranted to determine osmotic limitations and therefore the influence on native fish populations (including Delta Smelt) through predator–prey interactions.

### Thermal safety margins

Thermal safety margins (TSM), the difference between a fish’s upper temperature limits (CTMax values from 20°C-acclimated fish) and habitat temperatures differed by species and were dependent on location within the SFE. Non-native Silversides and Largemouth Bass had larger TSMs (5–14°C) compared to native Delta Smelt (3.6–8.6°C) at all SFE regions included in the analysis (Fig. 1), driven by the lower upper thermal tolerance limits of Delta Smelt (Table 3). Currently, wild Delta Smelt caught around their mean habitat temperature of 20°C may have a wide TSM of 8.6°C; however, average water temperatures in the SFE have been continuously increasing above 20°C over the past 20 years (see Fig. 1 in Jeffries *et al.*, 2016), are projected to increase in the SFE over the next 100 years (Brown *et al.*, 2013), and during drought periods and heat wave events temperatures routinely reach 25–28°C (Wagner *et al.*, 2011; Interagency Ecological Program MAST, 2015). Increasing periods of high temperature in the SFE is concerning as numerous studies indicate that climate change extinctions of species will occur in species with the narrowest TSM and demonstrating a limited capacity to acclimate (Stillman, 2003; Deutsch *et al.*, 2008; Gunderson and Stillman, 2015). Climate change predictions of 2–4°C increased in water temperature (Cloern *et al.*, 2011; Wagner *et al.*, 2011; Brown *et al.*, 2013) indicate Delta Smelt TSMs of less than 1°C at South Delta regions may reflect what TSMs of Delta Smelt could be across all SFE regions in the future. As previously described, Largemouth Bass do have further capacity to increase their upper thermal limits by 3–4°C above what was recorded in this current study (Smith and Scott, 1975; Currie *et al.*, 1998), thereby further increasing their TSM across all SFE regions. Delta Smelt may not have much if any additional acclimation capacity to increase upper thermal limits, although this requires investigation. Although upper thermal tolerance does vary across ontogeny, creating stage-specific TSMs, Komoroske *et al.* (2014) estimated TSMs for Delta Smelt finding negative margins in some cases for juveniles and adults compared to earlier larval stages with higher upper temperature tolerances. In evaluating implications of warming and increased salinity regimes on CTMax, it is important to acknowledge that CTMax (used to calculate TSM) provides insight into the maximum temperatures a fish can acutely survive and not how physiological performance is affected by sub-lethal warming on more ecologically relevant time scales. For example, Komoroske *et al.* (2015) showed Delta Smelt experience sub-lethal critical thresholds at 4–6°C below their CTMax such that fish had reduced ability to restore homeostastic mechanisms. Narrow TSM, limited physiological acclimation capacity, sub-lethal sensitivity, and already low population abundance (Hobbs *et al.*, 2017) suggest juvenile Delta Smelt will be particularly vulnerable to continued rises in Delta water temperatures and climate change.

In conclusion, each SFE species had the capacity to tolerate a series of multiple stressors that might be experienced during drought conditions. For prey species including Delta Smelt and Mississippi Silversides, short-term thermal history, but not salinity history influenced upper thermal tolerance; however, both thermal and salinity history influenced Largemouth Bass upper temperature tolerance. As expected, non-native fish were more thermally tolerant than native Delta Smelt and differed in thermal plasticity. Species-specific differences in temperature sensitivity and tolerances likely underlie the habitat range constraints in Delta Smelt and may explain increasing abundance and niche expansion in the non-native species. Climate change and increased occurrence of drought will likely continue to have a significant role in the shifted species assemblages in the SFE, favoring non-native fishes (as described in Quiñones and Moyle, 2014; Mahardja *et al.*, 2017). For example, native species including Longfin Smelt (*Spirinchus thaleichthys*), Chinook salmon (*Oncorhynchus tshawytscha*), and Steelhead (*Oncorhynchus mykiss irideus)* also exhibit lowered physiological tolerances and/or plasticity compared to non-natives (Cech and Myrick, 1999; Muñoz *et al.*, 2015; Jeffries *et al.*, 2016); however, there are some native species that will not be as vulnerable demonstrated by higher tolerances (to warming or salinity). Restoration projects assisting the conservation of native species in California’s SFE, to be successful in the long-term, must find a solution to deter non-native species that have wider physiological tolerances of environmental conditions and threaten to occupy restored habitat. The larger issue for endangered Delta Smelt is how to create thermal refugia due to their low thermal tolerance and increasingly warmer temperatures across the SFE. One restoration option to consider is to provide increased downstream (seaward) habitat, thereby facilitating cooler temperatures for Delta Smelt, which may be less favorable to other non-native species.

## Supplementary Material

Supplementary Data
