# Supplementary material for: Sensitivities of an endemic, endangered California smelt and two non-native fishes to serial increases in temperature and salinity: implications for shifting community structure with climate change
Source: Conserv Physiol. 2019 Feb 18;7(1):coy076. doi: 10.1093/conphys/coy076 (PMC6387996; doi:10.1093/conphys/coy076)
Supplement: Supplementary Data [file coy076_supplementary_material.docx]

**Supplementary material**

**Table S1.** Regression table of growth parameters across acclimation time for each San Francisco Estuary fish species. Intercept and slope values for experimental stressors Warm/Sal (T_High_:S_Low_ 🡪 T_High_:S_High_) and Sal/Warm (T_Low_:S_High_ 🡪 T_High_:S_High_) are in deviation to the Control (T_Low_:S_Low_) with the standard error (SE), T-value (T), and p-value (P) for body condition factor, standard length, and mass. Asterisks indicate a difference (p<0.05) in slopes (i.e. growth across the 25-day exposures) between stressor regimes Warm/Sal or Sal/Warm from the Control.

| **Species & Parameter** | **Regime** | **Intercept** | **SE** | **T** | **P-value** | **Slope** | **SE** | **T** | **P-value** |
| --- | --- | --- | --- | --- | --- | --- | --- | --- | --- |
| ***Delta Smelt*** |  |  |  |  |  |  |  |  |  |
| Body Condition | Control | 0.739 | 0.019 | 37.986 | <0.0001 | -0.001 | 0.001 | -0.553 | 0.581 |
|  | Warm/Sal | -0.020 | 0.027 | -0.729 | 0.466 | 0.001 | 0.002 | 0.591 | 0.555 |
|  | Sal/Warm | -0.034 | 0.027 | -1.224 | 0.222 | 0.003 | 0.002 | 1.578 | 0.116 |
| Length (mm) | Control | 37.083 | 1.107 | 33.485 | <0.0001 | -0.015 | 0.070 | -0.209 | 0.835 |
|  | Warm/Sal | 1.355 | 1.566 | 0.865 | 0.388 | 0.088 | 0.099 | 0.888 | 0.375 |
|  | Sal/Warm | -0.363 | 1.566 | -0.232 | 0.817 | 0.136 | 0.099 | 1.384 | 0.168 |
| Mass (mg) | Control | 383.295 | 42.984 | 8.917 | <0.0001 | 0.144 | 2.705 | 0.053 | 0.958 |
|  | Warm/Sal | 26.088 | 60.789 | 0.429 | 0.668 | 3.950 | 3.826 | 1.033 | 0.303 |
|  | Sal/Warm | -25.008 | 60.789 | -0.411 | 0.681 | 5.845 | 3.826 | 1.528 | 0.128 |
| ***Mississippi Silversides*** | |  |  |  |  |  |  |  |  |
| Body Condition | Control | 1.098 | 0.025 | 43.407 | <0.0001 | -0.002 | 0.002 | -0.959 | 0.338 |
|  | Warm/Sal | 0.016 | 0.036 | 0.436 | 0.664 | -0.0001 | 0.002 | -0.036 | 0.972 |
|  | Sal/Warm | 0.0260 | 0.036 | 0.726 | 0.468 | -0.001 | 0.002 | -0.454 | 0.650 |
| Length (mm) | Control | 33.924 | 0.945 | 35.896 | <0.0001 | -0.003 | 0.059 | -0.055 | 0.956 |
|  | Warm/Sal | -2.028 | 1.337 | -1.517 | 0.1305 | 0.170 | 0.083 | 2.037 | 0.043* |
|  | Sal/Warm | -1.821 | 1.337 | -1.362 | 0.1745 | 0.165 | 0.084 | 1.970 | 0.050* |
| Mass (mg) | Control | 442.108 | 39.295 | 11.251 | <0.0001 | -0.852 | 2.459 | -0.347 | 0.729 |
|  | Warm/Sal | -80.383 | 55.592 | -1.446 | 0.1494 | 7.125 | 3.479 | 2.048 | 0.042* |
|  | Sal/Warm | -56.930 | 55.592 | -1.024 | 0.3068 | 6.518 | 3.479 | 1.874 | 0.062 |
| ***Largemouth Bass*** | |  |  |  |  |  |  |  |  |
| Body Condition | Control | 2.163 | 0.062 | 34.888 | <0.0001 | 0.005 | 0.004 | 1.172 | 0.242 |
|  | Warm/Sal | -0.039 | 0.088 | -0.449 | 0.654 | 0.006 | 0.006 | 1.035 | 0.302 |
|  | Sal/Warm | -0.042 | 0.088 | -0.478 | 0.633 | 0.004 | 0.006 | 0.720 | 0.472 |
| Length (cm) | Control | 20.841 | 0.433 | 48.17 | <0.0001 | 0.006 | 0.027 | 0.220 | 0.826 |
|  | Warm/Sal | -0.651 | 0.612 | -1.063 | 0.289 | 0.032 | 0.039 | 0.847 | 0.398 |
|  | Sal/Warm | -0.295 | 0.612 | -0.482 | 0.63 | -0.004 | 0.039 | -0.114 | 0.910 |
| Mass (g) | Control | 199.495 | 12.794 | 15.593 | <0.0001 | 0.525 | 0.805 | 0.651 | 0.515 |
|  | Warm/Sal | -21.408 | 18.093 | -1.183 | 0.238 | 1.485 | 1.139 | 1.304 | 0.193 |
|  | Sal/Warm | -10.513 | 18.093 | -0.581 | 0.562 | 0.186 | 1.139 | 0.164 | 0.870 |
